# Advances in Wearable Biosensors for Non-Invasive Biofluid Monitoring

**DOI:** 10.3390/bios16060336

**Published:** 2026-06-14

**Authors:** Rajib Mondal, Manob Jyoti Saikia

**Affiliations:** 1 Electrical and Computer Engineering Department, University of Memphis, Memphis, TN 38152, USA; 2 Biomedical Sensors & Systems Lab, University of Memphis, Memphis, TN 38152, USA

**Keywords:** wearable biosensors, non-invasive sensing, biofluid analysis, precision healthcare, continuous monitoring, flexible electronics, human digital twins, point-of-care diagnostics, wireless health monitoring

## Abstract

Chronic diseases such as cardiovascular disorders, diabetes, neurological conditions, and kidney disease continue to rise worldwide. These conditions create a growing demand for continuous, non-invasive, and personalized health monitoring technologies. Wearable biosensors meet this need by enabling real-time physiological and biochemical measurements outside traditional clinical settings. Among wearable biosensors, those based on biofluids like sweat, tears, and saliva provide a painless alternative to blood sampling. These fluids also grant access to metabolites, electrolytes, hormones, proteins, and disease related biomarkers that reflect systemic health status. Advanced sensing technology allow us to continuously track health status by analyzing key biomarkers in these accessible biofluids. This review summarizes recent advances in non-invasive wearable biosensors and focuses on their sensing principles which includes biorecognition elements, signal transduction mechanisms, and data acquisition strategies. We also discussed key sensing modalities, including electrochemical, optical, thermal, and piezoelectric approaches, highlighting their advantages for wearable integration and performance in biofluid sensing. Finally the review also outlines recent developments and applications of these systems in biofluid sensing. In the end we highlights existing challenges, potential solutions, and future directions toward clinically deployable, AI-assisted precision healthcare systems.

## 1. Introduction

The global prevalence of chronic diseases, including cardiovascular disorders, diabetes, neurological conditions, and chronic kidney disease, continues to increase and places a major burden on healthcare systems worldwide. Cardiovascular health (CVH) remains a growing public health concern in the United States, with the American Heart Association projecting increases in hypertension from 51.2% to 61.0%, diabetes from 16.3% to 26.8%, obesity from 43.1% to 60.6%, and inadequate sleep from 40.3% to 42.1% among adults by 2050, although hypercholesterolemia, inadequate physical activity, poor diet, and current smoking are projected to decline [1]. Diabetes also represents a major global health burden, affecting approximately 589 million adults aged 20–79 years in 2024, corresponding to 11.11% of the global adult population and is projected to affect 853 million adults by 2050 [2]. Similarly, neurological disorders and chronic kidney disease continue to increase the global healthcare burden, affecting large populations and requiring long-term monitoring and management [3,4].

**Figure 1:**
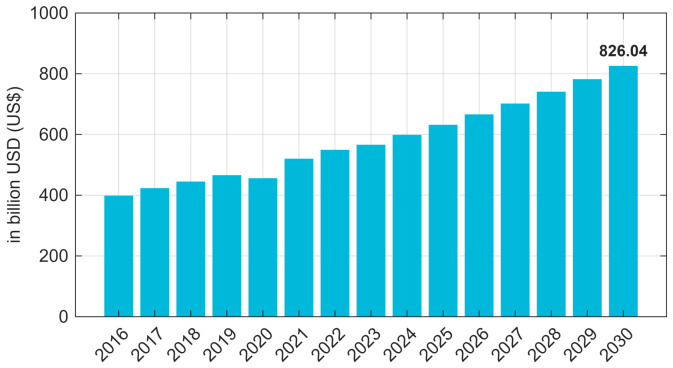
Global market size of wearable medical technologies.

This growing disease burden highlights the need for continuous, real-time, and non-invasive health monitoring technologies that can support early detection and timely intervention beyond traditional clinic-centered care. The demand for advanced medical technologies has therefore increased rapidly, and the global medical technology market is projected to exceed $800 billion by 2030 [5]. Figure 1 illustrates the market size and projected growth of wearable and medical monitoring technologies.

Motivated by these clinical and technological needs, non-invasive biosensing has emerged as an important direction for next-generation healthcare monitoring. Biofluids such as sweat, saliva, tears, and wound exudates provide an attractive alternative to conventional blood-based testing because they can be collected more easily and with less discomfort. These biofluids contain biomarkers such as metabolites, electrolytes, proteins, hormones, and pathogens, which can reflect physiological and pathological conditions. For example, sweat-based sensing enables the detection of glucose, chloride, uric acid, and creatinine for non-invasive assessment of diabetes, cystic fibrosis, and renal disorders [6]. Saliva- and tear-based platforms have also shown potential for rapid disease detection using chromatography, laser-induced fluorescence, electronic-nose systems, and advanced optical sensing methods [7,8]. In addition, sweat-based detection of opioid metabolites demonstrates the broader potential of biofluid sensing for healthcare and wellness monitoring [9].

The development of wearable biofluid biosensors has been supported by major advances in materials science, microfluidics, wireless communication, and data analytics. Flexible and stretchable substrates, including polydimethylsiloxane (PDMS) and hydrogels, together with nanomaterials such as graphene and carbon nanotubes, have improved sensor sensitivity, mechanical durability, miniaturization, and conformal contact with the body [10]. Microfluidic architectures and wireless communication technologies, such as Bluetooth and near-field communication (NFC), further enable controlled sampling, real-time data transmission, and portable health monitoring [11]. More recently, artificial intelligence (AI)-based data analytics has helped transform wearable biosensors into intelligent monitoring systems by supporting the interpretation of complex biosensing data and enabling personalized health decisions [12].

From a clinical perspective, saliva-, sweat-, and tear-based biosensors are useful only when their measured biomarker values can be interpreted in relation to established blood, plasma, serum, or clinically accepted reference measurements. Although these biofluids offer attractive non-invasive sampling routes, their analyte concentrations should not be assumed to directly represent circulating blood levels. Biomarker transport from blood to peripheral biofluids is analyte-specific and may be affected by secretion rate, dilution, local metabolism, evaporation, contamination, and sampling method. Therefore, wearable biofluid biosensors must demonstrate not only analytical sensitivity and selectivity, but also clinically meaningful performance metrics, including limit of detection, linear detection range, response time, signal stability, reproducibility, recovery, and agreement with standard laboratory assays.

Several recent studies show why performance-based evaluation is essential. Sweat cortisol sensing has been validated against ELISA with a high correlation coefficient of r = 0.973, and the sensor retained more than 90 percent of its response after 7 days of storage at 4 °C [13]. Tear glucose monitoring using the NovioSense conjunctival fornix sensor was evaluated in 24 patients with type 1 diabetes and reported a mean absolute relative difference of 16.7, a median absolute relative difference of 13.3, and 99.7 percent of values within Clarke error grid A+B regions after calibration to blood-equivalent glucose values [14]. Similarly, laser-engraved graphene sweat sensors achieved detection limits of 0.74 μM for uric acid and 3.6 microM for tyrosine, with sensitivities of 3.50 microA per microM per cm^2^ and 0.61 microA per microM per cm^2^, respectively [15]. These examples show that analytical performance alone is not sufficient; wearable biofluid biosensors must also demonstrate biofluid–blood correlation, clinical accuracy, long-term stability, and reproducibility under real sampling conditions.

Despite rapid progress, several challenges continue to limit the clinical translation of wearable biofluid biosensors. These include calibration drift, biofouling, sweat-rate or tear-flow variation, user-to-user variability, motion artifacts, sensor aging, and lack of standardized sampling and validation protocols. Saliva-based sensors may also be affected by diet, pH, oral bacteria, and salivary flow, while tear-based sensors must consider blinking, irritation, evaporation, and contact lens comfort. Therefore, paired biofluid–blood validation, standardized sampling protocols, clinically meaningful calibration models, and transparent reporting of numerical performance metrics are essential before these devices can be used for reliable diagnosis, disease monitoring, or treatment decision-making [14,16,17,18].

**Figure 2:**
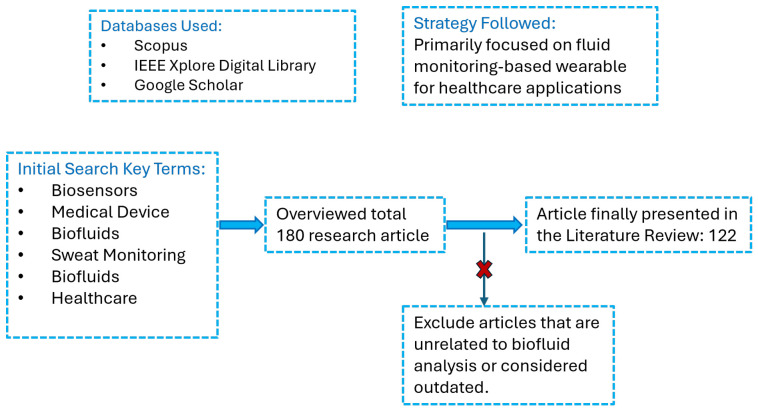
Schematic representation of the proposed review process, focusing on the literature concerning biofluids and their applications in healthcare.

Unlike many existing reviews that mainly summarize wearable biosensor designs, biofluid types, and sensing mechanisms, this review focuses on the translational readiness of non-invasive wearable biosensors for disease-related biofluid monitoring. The distinctive contribution of this review is its critical synthesis of sensor performance, material-device trade-offs, real-sample validation, calibration challenges, biofouling effects, long-term stability, and clinical applicability. Rather than presenting wearable biosensors only as emerging diagnostic tools, this review evaluates how effectively these systems can move from laboratory prototypes toward real-world and clinical use. Recent progress in advanced nanomaterials, microfluidic sampling, wireless integration, self-powered platforms, and AI-assisted data interpretation is also discussed from the perspective of practical deployment and clinical translation. Figure 2 summarizes the literature collection strategy and the scope of this review.

The main contributions of this review are summarized as follows:

1. It provides a critical synthesis of recent non-invasive wearable biosensors for biofluid-based disease monitoring.
2. It compares key sensor performance factors, including sensitivity, limit of detection, stability, selectivity, real-sample validation, and practical limitations.
3. It discusses major barriers to clinical translation, including calibration drift, sweat-rate variability, biofouling, user-to-user variability, long-term reliability, and lack of standardized validation.
4. It highlights recent advances in nanomaterials, microfluidic collection systems, wireless communication, self-powered sensing, and AI-assisted interpretation.
5. It identifies remaining research gaps and future opportunities for clinically reliable, scalable, and user-friendly wearable biosensing systems.

Overall, wearable biofluid biosensors have strong potential to support predictive, preventive, and personalized healthcare. However, their clinical value depends on reliable sensing performance, standardized sampling, stable calibration, and validation against accepted reference methods. By combining biofluid-based sensing with advanced materials, intelligent system design, and data-driven analytics, these technologies may become practical tools for continuous health monitoring in everyday life [19,20].

## 2. Working Principle of Biosensors

**Figure 3:**
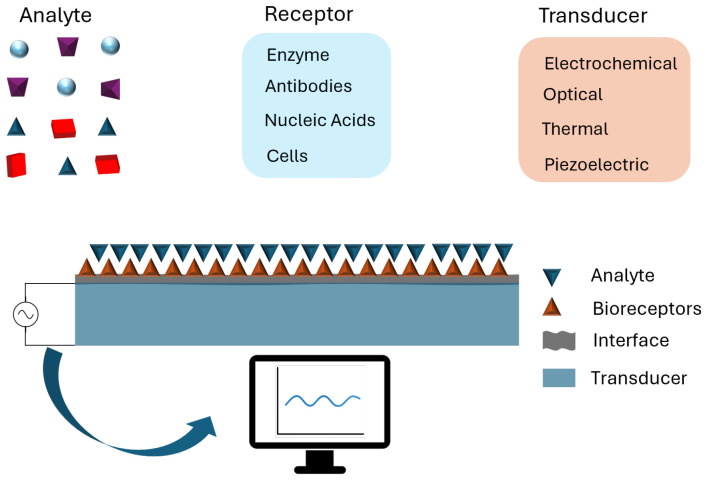
Schematic representation of biosensor operation principles, illustrating target analyte detection by the corresponding receptor molecule, followed by signal transduction and output.

Biosensors are analytical devices that convert biological interactions into measurable signals. A typical biosensor consists of three main components: a biorecognition element that selectively interacts with the target analyte, a transducer that converts this interaction into a physical signal, and a data acquisition or processing unit that records and interprets the output [21]. In wearable biofluid monitoring, this general structure must be adapted to flexible materials, low-power electronics, miniaturized sensing interfaces, and reliable operation under real-world conditions. Figure 3 illustrates the basic working process of a biosensor.

For non-invasive biochemical monitoring, wearable biosensors commonly analyze biofluids such as sweat, saliva, tears, interstitial fluid, and wound exudates. These biofluids can provide useful biochemical information, but their clinical interpretation requires careful validation because their analyte concentrations may not directly match blood values [16]. Representative examples include mouthguard biosensors for salivary glucose and uric acid monitoring, pacifier-based saliva sensors for infant glucose monitoring, eyeglass-integrated sweat sensors for electrolytes and metabolites, tear glucose sensors, and intelligent wound dressings for wound microenvironment monitoring [22,23,24,25,26,27]. These platforms show that the same biosensing principle can be adapted to different biofluids and wearable formats.

### 2.1. Major Types of Biosensors

Biosensors can be classified according to their transduction mechanism. In wearable applications, the choice of transducer strongly affects sensitivity, power consumption, device size, response time, and integration with flexible electronics.

- **Electrochemical biosensors** measure changes in current, potential, or impedance caused by analyte–bioreceptor interactions. Their low power requirement, high sensitivity, and compatibility with miniaturized electronics make them widely used in wearable and implantable systems [28].
- **Optical biosensors** detect changes in absorbance, fluorescence, surface plasmon resonance, or other light-based signals. They are useful for label-free and non-invasive sensing, especially when combined with compact imaging or smartphone-based readout systems [29].
- **Field-effect transistor biosensors** convert biochemical recognition events into changes in channel conductance. Their high electrical sensitivity and miniaturization potential make them attractive for wearable and implantable health monitoring [30].
- **Piezoelectric biosensors** detect analyte binding through changes in resonant frequency caused by mass loading or surface mechanical changes. They support rapid and label-free detection of biomolecules [31].
- **Thermal biosensors** measure heat changes generated during biochemical or enzymatic reactions. This approach can provide quantitative detection without optical labels or complex electrochemical mediators [32].

### 2.2. Biorecognition Elements

Biorecognition elements determine the selectivity and sensitivity of biosensors. Common recognition elements include enzymes, antibodies, nucleic acids, aptamers, and molecularly imprinted polymers. In wearable biofluid biosensors, the selected recognition element must not only bind or react with the target analyte, but also remain stable under variable pH, temperature, ionic strength, mechanical deformation, and long-term exposure to complex biofluids [33].

Enzyme-based recognition is widely used because enzymes provide strong catalytic specificity and can convert target biomarkers into measurable electrochemical signals. Glucose oxidase, lactate oxidase, uricase, and horseradish peroxidase are common examples in wearable sensing. Glucose oxidase-based systems have been used in saliva, sweat, and tear biosensors, while uricase-based systems have been used for salivary uric acid monitoring [22,23,26,34]. Enzymatic sensors are also used in wearable formats such as pacifier-integrated platforms and sweat-sensing systems for metabolic and electrolyte monitoring [24,25]. However, enzyme-based systems may suffer from enzyme degradation, limited storage stability, pH sensitivity, and signal drift during long-term use.

#### 2.2.1. Enzyme Cofactors and Redox Mediators

In enzyme-based electrochemical biosensors, efficient electron transfer between the enzyme active site and the electrode is essential for sensitivity, response time, operating potential, and long-term stability. Some oxidoreductase enzymes require cofactors such as NAD+/NADH or NADP+/NADPH, while others contain tightly bound redox centers such as FAD/FADH2. For example, glucose oxidase and lactate oxidase use flavin-based redox chemistry, while many dehydrogenase-based sensors depend on NAD+ or NADP+ as electron acceptors. However, direct electron transfer is often inefficient because the redox center may be buried inside the enzyme structure.

To overcome this limitation, redox mediators are often used to shuttle electrons between the enzyme and the electrode. Common mediators include ferrocene derivatives, Prussian Blue, quinones, conducting polymers, and osmium- or ferrocene-based redox polymers. Ferrocene-based mediators support electron transfer at relatively low operating potentials, which can reduce interference from electroactive species such as ascorbic acid and uric acid. Prussian Blue is also widely used in oxidase-based sensors because it enables low-potential detection of H_2_O_2_ and improves selectivity. Redox polymers can further “wire” enzymes to the electrode surface by forming a conductive network around the enzyme. Although these mediator-based strategies improve signal strength and miniaturization, mediator leaching, toxicity, enzyme degradation, pH sensitivity, and long-term drift remain important challenges for wearable applications [35,36,37,38].

#### 2.2.2. Molecularly Imprinted Polymers

Molecularly imprinted polymers are synthetic recognition materials prepared by polymerizing functional monomers around a target template molecule. After template removal, the polymer contains binding cavities that are complementary to the target in size, shape, and chemical functionality. Compared with natural receptors, MIPs offer several advantages for wearable biosensors, including chemical stability, reusability, tunable selectivity, low cost, and compatibility with flexible electrochemical platforms [39,40]. These properties are useful for continuous biofluid monitoring, where the sensing layer must tolerate variable pH, temperature, sweat rate, ionic strength, and mechanical deformation.

A representative wearable MIP example is the sweat-based electrochemical biosensor reported by Wang et al. [41]. This system used laser-engraved graphene electrodes functionalized with metabolite-specific MIP recognition layers and redox-active reporter nanoparticles. The platform also integrated iontophoresis-based sweat induction, microfluidic sweat sampling, signal processing, calibration, and wireless communication. Unlike conventional sweat sensors that mainly target high-concentration analytes such as glucose, lactate, sodium, and potassium, this MIP-based platform enabled monitoring of trace-level metabolites and nutrients, including essential amino acids and vitamins, during rest and exercise. This example demonstrates how MIPs can expand wearable biosensing toward analytes that are difficult to monitor using enzyme-based approaches.

MIPs can also be combined with enzyme-based recognition to improve sensing performance. In hybrid systems, the enzyme provides catalytic specificity and signal amplification, while the MIP layer can improve target selectivity, preconcentrate the analyte near the electrode, reduce interference from structurally similar molecules, and protect the enzyme from nonspecific adsorption. This strategy is useful for complex biofluids such as sweat, saliva, and tears, where proteins, salts, metabolites, and contaminants can distort sensor response. However, MIP-based systems still face limitations such as incomplete template removal, nonspecific adsorption, slow analyte diffusion, polymer swelling, and possible reduction of enzyme activity in enzyme–MIP hybrids. Therefore, future wearable MIP sensors should report selectivity, response time, regeneration stability, long-term drift, real-sample validation, and correlation with clinical reference measurements.

### 2.3. Signal Transduction Mechanisms

After the target analyte interacts with the biorecognition element, the biochemical event must be converted into a measurable signal. The selected transduction mechanism directly affects sensor sensitivity, selectivity, power requirement, response time, calibration stability, and compatibility with wearable integration [42]. Electrochemical transduction is one of the most widely used mechanisms in wearable biosensors because it requires low power and can be integrated with flexible and miniaturized electrodes. In this method, biochemical reactions are converted into electrical signals, usually measured as current, voltage, or impedance changes [43,44]. For example, glucose oxidase-based sensors convert glucose oxidation into an electrochemical signal through H2O2 generation or mediator-assisted electron transfer, while uric acid sensors rely on electrochemical oxidation reactions for metabolic monitoring [45,46,47]. However, electrochemical sensors may suffer from interference from other electroactive compounds, electrode fouling, signal drift, and the need for stable calibration. Optical transduction detects analyte recognition through changes in absorbance, fluorescence, chemiluminescence, surface plasmon resonance, optical waveguides, or photonic crystal structures [48]. Wearable optical biosensors often use colorimetric or fluorescence-based sensing layers, where analyte-responsive dyes produce visible spectral changes that can be captured by portable imaging systems such as smartphone cameras [49]. More advanced optical platforms use plasmonic nanostructures, nanopillars, or gold nanomesh architectures to improve sensitivity through surface plasmon resonance or surface-enhanced Raman scattering [50]. Although optical systems are attractive for non-invasive readout, they can be affected by lighting conditions, optical alignment, motion, sample turbidity, and calibration drift. Thermal transduction measures the heat generated or absorbed during biochemical reactions using temperature-sensitive elements such as thermistors, thermopiles, or enzyme thermistors [51,52]. This approach is useful because many biological reactions release or absorb heat, allowing for detection without optical labels or electrochemical mediators, and it has been applied to metabolites such as glucose, urea, and cholesterol [53]. However, wearable thermal biosensors must address heat dissipation, environmental temperature variation, sensor miniaturization, and low signal magnitude. Piezoelectric transduction detects biomolecular interactions through changes in mass loading or viscoelastic properties at the sensor surface. The most common platform is the quartz crystal microbalance, where analyte binding causes a shift in resonance frequency [31]. These sensors can be functionalized with antibodies, aptamers, or enzymes for label-free detection of cancer biomarkers, pathogens, viruses, toxins, and heavy metals [54]. Recent work has also explored nanomaterials and portable readout systems to improve signal amplification and point-of-care usability [55]. However, piezoelectric sensors still face challenges for wearable use because they are sensitive to mechanical disturbance, motion artifacts, and integration difficulties in flexible platforms.

### 2.4. Data Acquisition and Processing

After signal transduction, the biosensor output must be acquired, processed, and converted into meaningful health information. In wearable systems, this usually involves signal conditioning, amplification, filtering, baseline correction, analog-to-digital conversion, and wireless transmission to smartphones, smartwatches, or cloud platforms. Low-power communication technologies such as Bluetooth Low Energy, near-field communication, and Wi-Fi are commonly used depending on the device design and power budget [56]. Recent wearable biosensors increasingly integrate sensing, signal conditioning, processing, and wireless communication into compact and flexible platforms. Examples include saliva-based pacifier biosensors for infant glucose monitoring, mouthguard and contact lens glucose sensors, and eyewear-based systems for sweat metabolite monitoring [22,24,25]. The final stage involves data interpretation and user feedback through mobile applications or cloud-based dashboards. Machine learning and artificial intelligence can support pattern recognition, anomaly detection, adaptive calibration, and early warning of abnormal physiological trends [27]. However, AI-assisted interpretation requires high-quality data, representative validation, and transparent calibration to avoid unreliable predictions.

### 2.5. Calibration and Standardization Requirements for Wearable Biofluid Biosensors

Calibration is essential for translating wearable biofluid biosensors from laboratory prototypes to clinically reliable devices. Although factory calibration is feasible in mature systems such as some commercial continuous glucose monitors, it is more difficult for saliva-, sweat-, and tear-based biosensors because sensor output is affected by biofluid volume, flow rate, local chemistry, temperature, pH, contamination, sensor aging, and biofouling. Therefore, factory calibration alone may not be sufficient [57]. A more practical strategy may combine factory calibration, user-specific baseline correction, periodic reference checks, and adaptive algorithm-based recalibration. For sweat-based sensors, calibration is especially challenging because sweat composition changes with sweat rate, anatomical site, hydration status, exercise intensity, temperature, and collection method. Ion-selective sensors for sodium and potassium, for example, require careful calibration because potentiometric response can drift over time and can be influenced by ionic interference and changing sweat composition [17,57].

For saliva- and tear-based biosensors, user-specific calibration is also important because biomarker concentration can be affected by salivary flow, diet, oral contamination, tear-flow rate, blinking, evaporation, and local surface conditions [58]. In tear glucose monitoring, calibration models may be needed to convert tear sensor signals into blood-equivalent glucose values, as shown by the NovioSense tear glucose sensor using a neural-network-based calibration model [14]. Sensor drift and biofouling remain major barriers because proteins, salts, lipids, dead cells, and environmental contaminants can accumulate on the sensor surface, reduce sensitivity, shift the baseline, and slow response time [33]. Therefore, future wearable biosensors should report calibration conditions, recalibration frequency, drift over time, storage stability, on-body stability, response recovery, and resistance to biofouling. Low-burden calibration strategies, such as short user-specific initialization, automatic baseline tracking, built-in quality-control checks, and algorithmic correction for temperature, pH, sweat rate, and signal drift, are necessary to improve reliability, comparability, and clinical acceptance [16,17,59].

## 3. Sweat-Based Biosensors

Sweat has emerged as one of the most promising biofluids for non-invasive and continuous health monitoring using wearable biosensors. Unlike blood sampling, which is invasive and often painful, sweat can be collected directly from the skin surface with minimal discomfort and without the need for trained personnel [60]. This ease of access, combined with advances in wearable sensor technologies, has positioned sweat as a highly attractive medium for real-time physiological and biochemical monitoring [61,62].

Sweat contains a broad spectrum of biomarkers that reflect the body’s physiological and metabolic state. Major constituents include electrolytes such as Na^+^, K^+^, and Cl^−^, typically present in concentrations ranging from 10 to 100 mM, which provide insight into hydration status, electrolyte balance, and thermoregulation [63]. In addition, sweat carries metabolites such as glucose, lactate, urea, uric acid, and ammonia, which are closely associated with energy metabolism, renal function, and physical exertion. These biomarkers enable continuous monitoring of metabolic activity during daily living and physical performance. The detection of biochemical substances in sweat presented in this review is listed in Table 1.

Beyond electrolytes and metabolites, sweat also contains hormones and signaling molecules that support broader health assessment. Hormones such as cortisol, dehydroepiandrosterone (DHEA), and neuropeptide Y (NPY) have been detected in sweat and are linked to stress response, fatigue, and neuroendocrine regulation. Notably, sweat cortisol levels, which are approximately one-tenth of corresponding plasma concentrations, have been shown to reliably track the free, biologically active fraction of cortisol in blood [64]. Furthermore, proteins and cytokines including IL-6, IL-31, TNF-alpha, IFN-gamma, and Annexin-5 have been identified in sweat, reflecting immune activity and inflammatory processes. Disease-associated proteins such as Bleomycin Hydrolase and Synaptophysin further highlight the proteomic diagnostic potential of sweat [65].

Recent advances in sweat-based biosensing have expanded detectable targets to include nutrients and micronutrients. Wearable platforms capable of sensing compounds such as vitamin C have been reported, broadening the application of sweat analysis to nutritional assessment and metabolic health monitoring [66]. Collectively, continuous sweat analysis offers a non-invasive pathway for real-time evaluation of hydration status, stress levels, metabolic disorders, and immune function. These capabilities position sweat-based biosensors as a powerful tool for personalized healthcare, preventive medicine, and human performance management [61,63].

**Table 1:** Summary of the detection mechanism of biochemical substances in sweat.

Analytes	Detection Methods	Recognition Elements	Preparation Technologies	Related Diseases	Application Area	References
Na^+^	OCPT	Na^+^ ionophore	Screen printing; laser engraving	Hypernatremia; hyponatremia	Outdoor monitoring	[67,68]
Cl^−^	Colorimetry	Silver chloranilate; silver chlorobenzoate	CO_2_ laser manufacturing; roll-to-roll printing	Cystic fibrosis	Outdoor monitoring	[69,70]
K^+^	OCPT	K^+^ ionophore	Screen printing; laser engraving	Hyperkalemia; hypokalemia	Outdoor, family monitoring	[69,70]
Glucose	CA	Glucose oxidase	Roll-to-roll printing; laser engraving; screen printing	Diabetes; hypoglycemia	Disease monitoring	[71]
Lactate	Colorimetry; CA	Lactate oxidase	Inkjet printing; laser engraving; screen printing	Hypoglycemia; hyperlactatemia	Family monitoring	[70,72]
Uric acid	CA	Not available	Laser engraving	Gout; uric acid stones	Family monitoring	[71]
Ascorbic acid	DPV	Ascorbic acid oxidase	Laser engraving; screen printing; photolithography and evaporation	Scurvy	Family monitoring	[72,73]
L-Dopamine	DPV	Not available	Laser engraving	Parkinson’s disease	Drug monitoring	[74]
Tyrosine	DPV; SWV; CV	SilkNCT	Photolithography and electron beam evaporation	Tyrosinemia	Health monitoring	[15]
Caffeine	DPV	Multiwalled carbon nanotubes	Laser engraving	Not available	Not available	[71,75]
Cortisol	CV	Cortisol antibody	Roll-to-roll printing; screen printing	Depressive disorder	Disease monitoring	[76]
Nicotine	Not available	Not available	Photolithography and electron beam evaporation	Nicotine poisoning	Drug monitoring	[77]

Detection method abbreviations: OCPT = Open Circuit Potential Technique; CA = Chronoamperometry; DPV = Differential Pulse Voltammetry; SWV = Square Wave Voltammetry; CV = Cyclic Voltammetry

### 3.1. Sweat Collection Strategies

Understanding sweat generation and transport is essential for the accurate design and interpretation of sweat-based biosensing systems. The majority of measurable sweat is produced by eccrine sweat glands, which are widely distributed across the human body. Each eccrine gland consists of a secretory coil, where sweat is initially generated, and a dermal duct that transports the fluid to the skin surface. During sweat formation, a variety of analytes including ions, metabolites, organic acids, hormones, and small proteins are transferred from surrounding blood vessels and interstitial fluid into the sweat matrix [78]. Sodium (Na^+^) and chloride (Cl^−^) are the dominant ionic constituents of sweat and play a critical role in the secretion process. Active ion transport within the secretory coil establishes an osmotic gradient that drives water influx into the gland lumen. As sweat travels through the dermal duct, partial reabsorption of Na^+^ and Cl^−^ occurs via epithelial ion channels. Because the rate of ionic reabsorption remains relatively constant, the final concentrations of Na^+^ and Cl^−^ in secreted sweat increase proportionally with sweat rate, directly linking sweat composition to physiological and environmental conditions [79].

From a sensing perspective, sweat can be collected using either passive or active strategies. Passive sweat collection relies on natural perspiration during thermoregulation, physical activity, or environmental heat exposure and is well suited for continuous, real-world monitoring. In contrast, active sweat stimulation techniques are employed to induce sweat secretion under controlled conditions, improving sample availability and reproducibility, particularly in clinical or low-sweat scenarios. The selection of a sweat collection strategy directly influences analyte concentration, temporal resolution, and system reliability, and therefore plays a crucial role in determining the suitability of wearable sweat biosensors for continuous monitoring, point-of-care diagnostics, and personalized healthcare applications [80].

#### 3.1.1. Passive Collection (Natural Perspiration)

Passive sweat collection relies on spontaneous perspiration generated through natural thermoregulation, typically induced by physical activity or elevated ambient temperature. This approach is attractive due to its simplicity and minimal system complexity, as it does not require external stimulation or additional power, and it preserves the physiological relevance of naturally secreted sweat [80]. As a result, passive collection is well suited for continuous, real-world monitoring applications, particularly in sports, fitness tracking, and daily health assessment.

In wearable microfluidic platforms, passive sweat collection typically achieves sweat rates on the order of 0.79 mL per minute [81]. However, both sweat rate and composition can vary substantially across individuals and environmental conditions, including temperature, humidity, eccrine gland density, hydration status, and physical fitness. These variations introduce uncertainty in analyte concentration and temporal resolution, which can complicate quantitative interpretation and cross-subject comparison. Another limitation of passive sweat collection is the delayed onset of stable sweat secretion. Under moderate exercise conditions, steady-state sweating often requires 20 to 30 min to initiate, which restricts its applicability for sedentary users, resting-state measurements, or time-sensitive clinical diagnostics [82]. Consequently, while passive sweat collection offers a non-invasive and user-friendly strategy, its dependence on physiological and environmental factors necessitates careful system design and calibration for reliable wearable biosensing.

#### 3.1.2. Active Stimulation Methods

Active sweat stimulation methods are used to induce sweat under controlled conditions, especially when natural sweating is low or when standardized sampling is needed. Compared with passive collection, active stimulation can improve control over sweat onset, sampling location, and collection timing, which is useful for clinical diagnostics and wearable biosensing studies [16].

**Figure 4:**
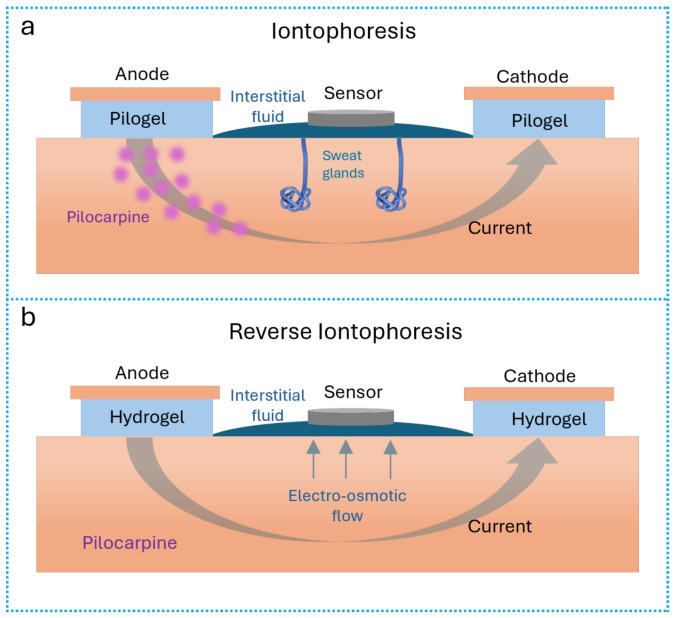
Iontophoresis and reverse iontophoresis process: (**a**) Iontophoresis relies on topical current application for local sweat stimulation. (**b**) Reverse iontophoresis uses current application to extract analytes electro-osmotically. Adapted from [84].

Iontophoresis is the most widely used active sweat stimulation method. As shown in Figure 4a, a mild electrical current, typically about 1 to 2 mA for approximately 5 min, is applied to deliver cholinergic agents such as pilocarpine nitrate through the skin [83]. Pilocarpine activates muscarinic receptors in eccrine sweat glands and induces localized sweat secretion without requiring exercise. Wearable iontophoretic systems commonly combine stimulation electrodes, drug-loaded hydrogels, microfluidic collection layers, and electrochemical sensors for on-body sweat induction and real-time biochemical analysis [82]. Temporary tattoo-based platforms have also demonstrated combined sweat stimulation and electrochemical sensing for applications such as ethanol and glucose monitoring, showing the potential of iontophoresis for compact and user-friendly wearable devices [79].

Despite these advantages, iontophoresis has limitations. Repeated or prolonged stimulation at the same skin site may cause irritation, erythema, or discomfort. In addition, differences in skin impedance, drug delivery efficiency, and individual sweat-gland response can affect sweat rate and analyte concentration, requiring careful calibration and protocol optimization [83]. Reverse iontophoresis, shown in Figure 4b, uses electro-osmotic transport to extract interstitial fluid rather than stimulate sweat secretion and has been explored for non-invasive glucose monitoring [85]. However, continuous electro-stimulation can reduce comfort and user compliance. Therefore, future active stimulation systems should focus on lower stimulation intensity, improved skin-compatible materials, reliable calibration, and integration with microfluidic control for long-term wearable use.

#### 3.1.3. Passive Versus Active Sweat Collection

Passive, exercise-induced, and iontophoresis-induced sweat samples should not be assumed to have identical composition. Sweat composition is affected by sweat rate, anatomical site, gland stimulation pathway, skin surface contamination, collection duration, evaporation, and ductal reabsorption of electrolytes. Passive sweat collection may better reflect natural sweating, but it is often limited by low sample volume, longer collection time, and contamination from skin residues. Exercise-induced sweat provides larger sample volume but can be strongly influenced by body temperature, exercise intensity, hydration status, metabolic activity, and environmental conditions. Iontophoresis-induced sweat, commonly stimulated using pilocarpine, offers more controlled local sweat generation and is widely used in sweat chloride testing, but pharmacological stimulation may not fully represent natural sweat physiology. For example, Vairo et al. reported no correlation between exercise-induced sweat electrolytes and plasma electrolytes, while pilocarpine-induced sweat showed a correlation only for potassium with plasma potassium (R = 0.78) [86]. They also observed higher intra-individual variability in exercise-induced sweat compared with pilocarpine-induced sweat. Similarly, Souza et al. compared pilocarpine-induced, exercise-induced, and passively collected sweat using metabolomic analysis and showed that collection method can affect sweat metabolite profiles and repeatability [87]. Therefore, wearable sweat biosensor studies should clearly report whether sweat was collected passively, by exercise, by thermal stimulation, or by iontophoresis, and direct comparison across collection methods should be avoided unless method-specific calibration and validation are performed [88].

#### 3.1.4. Microfluidic and Absorbent-Based Collection

Modern wearable sweat biosensing platforms increasingly employ microfluidic and absorbent-based architectures to enable controlled sweat capture, transport, and analysis. Microfluidic systems incorporate enclosed channel networks that guide sweat from the skin surface to designated sensing regions, allowing precise regulation of sample flow while minimizing evaporation, contamination, and analyte loss [79,82]. This level of control is particularly critical under low sweat-rate conditions, where accurate temporal resolution and concentration-dependent measurements are required for reliable biomarker quantification.

Absorbent-based collection strategies, typically implemented using hydrogels, porous membranes, paper substrates, or textile materials, complement microfluidic designs by stabilizing small sweat volumes and maintaining consistent skin contact [58]. These materials act as passive reservoirs that buffer fluctuations in sweat secretion, improve sample retention, and support prolonged monitoring. In addition, absorbent layers can be engineered to selectively filter particulates or delay analyte transport, enabling time-resolved or sequential sweat analysis [89].

The integration of microfluidic routing with absorbent collection layers has enabled compact, skin-conformal wearable platforms compatible with electrochemical, colorimetric, and optical sensing modalities. Such hybrid systems facilitate continuous and quantitative monitoring of electrolytes, metabolites, and stress-related biomarkers in real time [82,90]. Overall, microfluidic and absorbent-based sweat collection strategies play a critical role in improving sampling efficiency, analytical accuracy, and long-term usability of wearable sweat biosensors, supporting their deployment in personalized healthcare and point-of-care diagnostics.

### 3.2. Skin–Device Interface and Biofluid Sampling Standardization

The skin–device interface strongly affects the reliability of wearable biofluid biosensors. For sweat-based devices, the patch must maintain conformal skin contact while allowing fresh sweat to reach the sensing area. Poor contact, motion, weak adhesion, and skin deformation can cause unstable signals, delayed sampling, and inaccurate biomarker readings. At the same time, excessive adhesion or low breathability may cause irritation, discomfort, sweat blockage, and poor long-term wearability. Therefore, flexible wearable sensors should be evaluated not only by analytical performance, but also by skin compatibility, adhesive stability, breathability, mechanical robustness, and comfort during movement [33,78,91].

Sweat sampling also requires careful standardization because evaporation, contamination, sweat-rate variation, and old sweat accumulation can change the apparent concentration of biomarkers. Skin oils, cosmetics, dead cells, bacteria, and environmental residues may introduce additional interference. Microfluidic sweat collection can reduce these problems by guiding fresh sweat to the sensor, limiting evaporation, separating old and new sweat, and enabling sweat-rate monitoring [88,92,93]. However, microfluidic systems may also introduce dead volume, delayed transport, channel blockage, and limited operation time. Thus, wearable sweat studies should report the sweat induction method, anatomical site, sweat rate or volume, collection time, sensor contact area, environmental condition, and reference validation method.

For saliva- and tear-based biosensors, sampling conditions are also critical. Saliva composition can be affected by diet, hydration, oral hygiene, bacterial activity, pH, salivary flow rate, and time of day. Therefore, oral biosensor studies should report whether measurements were taken under fasting, post-meal, resting, or stimulated conditions [18,94]. Tear-based devices must additionally consider tear volume, evaporation, blinking, ocular irritation, lens movement, oxygen permeability, and optical transparency [95,96,97]. Overall, reliable clinical translation requires standardized sampling, interface stability, user comfort, and validation against accepted reference assays.

## 4. Application of Wearable Biosensors

### 4.1. Sweat Based Wearable Sensing

Sweat-based wearable biosensors have rapidly evolved from simple single-analyte devices into integrated, multiplexed systems capable of simultaneous biochemical and physiological monitoring. Recent studies increasingly report key sensing-performance metrics, including detection limit, sensing range, sensitivity, stability, response time, real-sample validation, and calibration reliability. These metrics are essential because sweat biomarkers are often present at lower and more variable concentrations than blood biomarkers, and sensor signals can be affected by sweat rate, evaporation, skin contamination, temperature, pH, motion, and biofouling.

**Figure 5:**
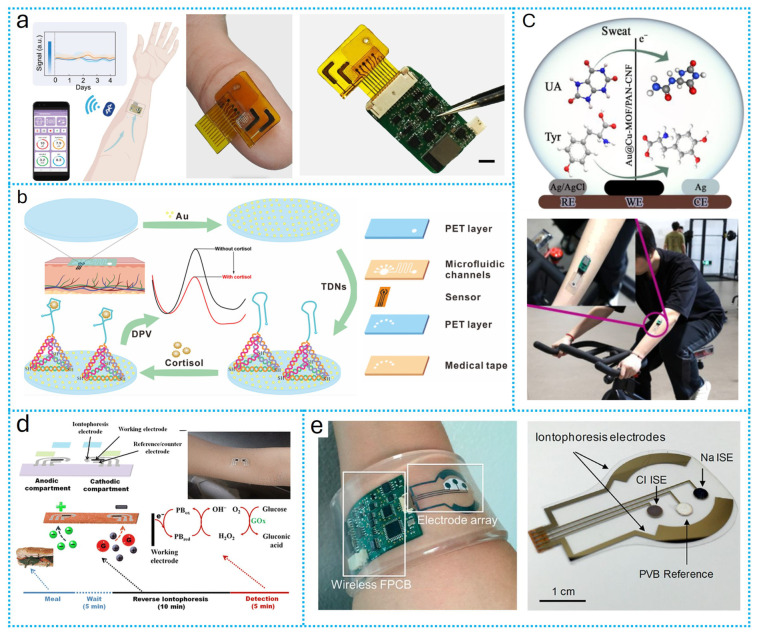
Sweat-based wearable biosensors: (**a**) Microfluidic wearable sensor for multiday sweat sampling and monitoring of sweat ingredients. Reproduced with permission [98]. (**b**) Laser-engraved microfluidic sensor for continuous non-invasive sweat cortisol monitoring. Reproduced with permission [99]. (**c**) Flexible electrochemical biosensor using Au@Cu-MOF/PAN-CNF electrodes for selective detection of uric acid and tyrosine during exercise. Reproduced with permission [100]. (**d**) Tattoo-based reverse iontophoretic glucose sensor for noninvasive on-skin glucose monitoring. Reproduced with permission [85]. (**e**) Iontophoretic wristband for simultaneous detection of chloride, sodium, and glucose in induced sweat. Reproduced with permission [83].

Recent bioinspired microfluidic systems have improved long-term sweat sampling by addressing inconsistent secretion, rapid evaporation, and dependence on exercise, as illustrated in Figure 5a. For example, the BMS3 platform integrated hierarchically graded microchannels, superhydrophobic–superhydrophilic Janus membranes, and a miniaturized iontophoresis module to enable low-volume sweat collection, autonomous sweat induction, and multiday metabolic monitoring of uric acid, xanthine, and alcohol [98]. From a sensing-performance perspective, the xanthine, uric acid, and alcohol sensors showed sensitivities of 0.37 nA μM^−1^ mm^−2^, 0.93 nA μM^−1^ mm^−2^, and 26.2 nA mM^−1^ mm^−2^, respectively. The integrated pH sensor showed a sensitivity of 59.5 mV pH^−1^, while the temperature sensor showed a relative sensitivity of 0.138% °C^−1^ over 25–45 °C. The enzymatic sensors also maintained storage stability for more than 4 weeks at 4 °C. These results show that BMS3 improves both sweat handling and biochemical detection, although larger clinical validation is still required to confirm whether long-duration sweat profiles can reliably represent systemic metabolic status.

Earlier multianalyte electrochemical platforms established the foundation for real-time, non-invasive tracking of key metabolites and electrolytes. A fully integrated patch-type sensor array enabled simultaneous monitoring of glucose, lactate, sodium, potassium, and skin temperature during exercise by coupling flexible electrodes with on-board signal conditioning, data processing, and wireless transmission circuitry [82]. The glucose and lactate sensors showed linear response over sweat-relevant ranges, while the Na^+^ and K^+^ sensors were tested over 10–160 mM and 1–32 mM, respectively. The reported sensitivities were 64.2 mV/decade for Na^+^ and 61.3 mV/decade for K^+^, which are close to the theoretical Nernstian response. The device also demonstrated stable biosensor performance over several weeks, supporting repeated wearable use. However, accurate interpretation still requires calibration because sweat rate, local temperature, and ion composition can change during exercise and influence the final sensor output.

Subsequent efforts extended this concept through microfluidic and soft electronic design. A laser-engraved wearable electrochemical aptasensor was developed for non-invasive sweat cortisol monitoring, as shown in Figure 5b. The sensor used gold nanoparticle-modified laser-engraved graphene electrodes with tetrahedral DNA nanostructures to improve conductivity and aptamer immobilization. Cortisol was detected through a methylene blue-based aptamer mechanism, where cortisol binding changed the electron-transfer distance between methylene blue and the electrode surface [99]. The reported analytical range was 1 pM/μM, with a low detection limit of 0.2 pM in sweat. On-body testing also demonstrated dynamic cortisol monitoring for up to 90 min. These values indicate strong sensitivity for low-abundance stress biomarkers, but sweat cortisol monitoring still faces challenges related to aptamer stability, non-specific adsorption, sweat-rate variation, and calibration against blood, saliva, or standard laboratory assays.

Another laser-engraved, skin-interfaced sensor was developed for simultaneous sweat sampling, chemical detection, and vital-sign monitoring. The device continuously measured temperature, respiration rate, and trace metabolites such as uric acid and tyrosine, enabling dynamic profiling of metabolic activity in both trained and untrained individuals [15]. The laser-engraved graphene chemical sensor showed high analytical performance for uric acid and tyrosine, with sensitivities of 3.50 μA μM^−1^ cm^−2^ and 0.61 μA μM^−1^ cm^−2^, respectively. The corresponding limits of detection were 0.74 μM for uric acid and 3.6 μM for tyrosine. The sensor also enabled direct detection in sweat and saliva and showed mechanical and electrochemical stability. Importantly, sweat uric acid levels were higher in gout patients than in healthy controls, and a similar trend was observed in serum. This supports the clinical potential of sweat-based uric acid monitoring, although larger cohorts are still needed to define diagnostic thresholds and inter-subject variability.

Current advances in materials engineering have further improved sensitivity and selectivity toward multiple analytes. A notable example is the composite electrode integrating gold nanoparticles with copper metal–organic-framework-derived carbon nanofibers, written as Au@Cu-MOF/PAN-CNF, for uric acid and tyrosine detection, as illustrated in Figure 5c [100]. The large surface area, tunable porosity, accessible active sites of the Cu-MOF structure, and improved electron transfer from gold nanoparticles and carbon nanofibers enhanced the electrochemical response toward target metabolites. The wearable patch also incorporated a lightweight circuit board for wireless transmission, supporting portable monitoring of human metabolism. The study reported selective and reproducible detection of uric acid and tyrosine and demonstrated continuous DPV-based monitoring during sweat-flow conditions. However, broader clinical testing, long-term on-body stability analysis, and direct comparison with reference biochemical assays remain necessary before this type of MOF-based patch can be considered clinically reliable. To enable sweat analysis independent of physical activity, iontophoretic and reverse-iontophoretic systems have been introduced. A tattoo-based glucose sensor employed reverse iontophoresis to extract interstitial fluid through electro-osmotic transport and quantify glucose using a compact skin-mounted electrode configuration, as illustrated in Figure 5d [85]. This approach is attractive because it reduces dependence on natural sweating; however, reverse iontophoresis may be affected by skin impedance, local pH, stimulation current, and user discomfort during repeated use. Similarly, an integrated iontophoretic wristband combined localized sweat stimulation and multiplexed sensing of chloride, sodium, and glucose in pilocarpine-induced sweat, as shown in Figure 5e [83]. The Na^+^ and Cl^−^ sensors showed near-Nernstian responses, with sensitivities of approximately 63.2 mV/decade and 55.1 mV/decade, respectively, while the glucose sensor showed a sensitivity of about 2.1 nA μM^−1^. These platforms allow controlled sweat generation without exercise, making them relevant for cystic fibrosis testing and glycemic monitoring. However, induced-sweat platforms still face limitations such as finite sampling duration, possible local skin irritation, variable pilocarpine response, and the need for calibration between local sweat composition and systemic biomarker levels.

Overall, sweat-based biosensors show strong potential for continuous health monitoring because they can combine non-invasive sampling, flexible electronics, microfluidics, wireless communication, and multimodal sensing. However, the field still faces important translational barriers. First, sweat biomarker concentrations may not always directly match blood levels. Second, sensor calibration can be affected by sweat rate, temperature, evaporation, and contamination from the skin surface. Third, long-term use may be limited by enzyme degradation, biofouling, adhesive failure, and skin irritation. Fourth, many studies are still validated only in small cohorts or controlled exercise settings. Therefore, future sweat-based biosensor studies should report standardized performance metrics, including limit of detection, linear range, sensitivity, response time, selectivity, storage stability, on-body stability, real-sample validation, sample size, and correlation with clinical reference methods. Such performance-based reporting will make the literature more comparable and strengthen the clinical translation of sweat-based wearable biosensing systems.

### 4.2. Tear-Based Biosensors

Tear-based wearable biosensors utilize the biomarker-rich composition of tears for noninvasive health monitoring, with a major focus on metabolites such as glucose for diabetes management, as well as proteins, electrolytes, and inflammatory markers for ocular disease diagnostics. Contact lens platforms dominate this area because they provide direct access to basal tears with minimal interruption to daily activity. However, the diagnostic interpretation of tear biomarkers remains challenging because tear volume, blinking rate, reflex tearing, evaporation, ocular irritation, and local inflammation can affect biomarker concentration and sensor output. Therefore, tear-based biosensors should be evaluated using clear performance metrics, including limit of detection, sensing range, sensitivity, response time, selectivity, mechanical stability, biocompatibility, and validation against blood or standard laboratory assays [96,101]. Tear-based wearable biosensors have advanced noninvasive monitoring by using the biochemical profile of tears, which can reflect systemic changes for selected metabolites and electrolytes. However, the correlation between tear and blood biomarkers is not always direct and may vary by analyte, subject, and sampling condition. Contact lens platforms provide continuous basal tear contact and can be fabricated from soft, oxygen-permeable materials to improve wearer comfort and sensing reliability [96]. Early optical sensors exploited glucose interactions with concanavalin A or phenylboronic acid for holographic or diffraction-based detection, enabling battery-free monitoring concepts [97]. Photonic crystal and hydrogel combinations incorporating fluorescent or diffractive sensing elements further expanded the detectable analyte range, while smartphone-based imaging allowed low-cost signal readout and data interpretation [102].

**Figure 6:**
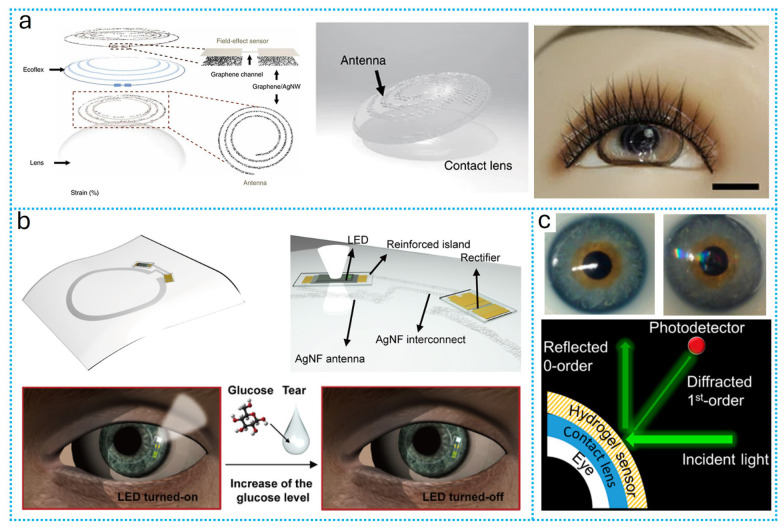
Representative tear-based wearable biosensors: (**a**) Smart contact lens integrating glucose and intraocular pressure sensors, enabling wireless in vivo glucose monitoring in a rabbit eye and in vitro pressure monitoring using a bovine eyeball. Reproduced with permission [101]. (**b**) Expanded smart contact lens for real-time visualization of tear glucose response in a rabbit model. Reproduced with permission [103]. (**c**) Hydrogel-based photonic microstructure sensor attached to a commercial contact lens for optical continuous glucose monitoring. Reproduced with permission [104].

More recently, smart contact lenses for wireless ocular diagnostics have integrated glucose and intraocular pressure sensors, demonstrating in vivo glucose monitoring in rabbits and in vitro pressure assessment in bovine eyeballs, as illustrated in Figure 6a [101]. Kim et al. used graphene and graphene-AgNW hybrid structures to achieve more than 91% optical transparency and approximately 25% stretchability, which are important for maintaining visual clarity and mechanical compatibility with soft contact lenses. The glucose sensor detected concentrations from 1 μM to 10 mM, with a signal-to-noise ratio of 7.34 at 1 μM and a calculated limit of detection of 0.4 μM. The sensor was also responsive within the typical tear glucose range of 0.1–0.6 mM and showed no major sensitivity degradation after storage in artificial tear solution for 24 h. For intraocular pressure monitoring, the resonance frequency decreased with increasing pressure, showing an approximately linear response from 5 to 50 mmHg with a sensitivity of 2.64 MHz/mmHg. These results demonstrate strong analytical and mechanical performance; however, the in vivo validation was limited to a rabbit model, and further human studies are required to confirm long-term comfort, calibration stability, and clinical accuracy. Further extensions have incorporated wireless power transfer and integrated displays, enabling real-time visualization of tear glucose responses without visual obstruction through transparent and soft materials, radio-frequency antennas, and miniaturized LEDs, as illustrated in Figure 6b [103]. Park et al. developed a soft smart contact lens integrating a glucose sensor, rectifier, wireless antenna, and LED display. The device was wirelessly powered, and the LED response was used as a visual indicator of glucose level change in tear fluid. This design is important because it moves beyond passive sensing and adds an on-lens display function. However, the display-based output is closer to a threshold-type warning than a fully quantitative continuous glucose monitoring system. Therefore, additional work is needed to improve quantitative calibration, long-term enzymatic stability, and human-subject validation under daily glucose fluctuations.

Another advancement involves smartphone-based optical glucose monitoring using a photonic hydrogel attached to commercial contact lenses. This approach measures changes in reflected light intensity in response to tear glucose levels, as shown in Figure 6c [104]. Elsherif et al. reported a glucose detection range of 0–50 mM, a sensitivity of 12 nm/mM, and a saturation response time of less than 30 min. The same study also reported a short response time of 3 s and a saturation time of 4 min in continuous monitoring mode. The sensor was integrated with commercial contact lenses and read using a smartphone camera, which makes the platform attractive because it reduces the need for complex miniaturized electronics and external power sources. However, the response time may still be relatively slow for some rapid glucose fluctuations, and the reported glucose range is broader than the typical physiological tear glucose range. Therefore, future optical contact lens sensors should be optimized for low-concentration tear glucose, faster response, stable optical calibration, and validation under real blinking and tear-flow conditions. Alternative designs include the NovioSense conjunctival fornix sensor for tear glucose monitoring. Unlike contact lens sensors that sit directly on the cornea, the NovioSense device is placed under the lower eyelid, where it can access tear fluid while reducing visual obstruction [26]. In a later clinical evaluation of 24 patients with type 1 diabetes, the sensor was worn for a total of 6 h, including 1 h of equilibration and 5 h of measurement. The study reported a mean absolute relative difference of 16.7 and a median absolute relative difference of 13.3 after applying a neural-network-based calibration model to convert tear glucose readings into blood-equivalent glucose values [14]. In addition, 99.7% of the data points were located in the Clarke error grid A+B regions, and no adverse events were attributed to the sensor coil. These results provide stronger clinical evidence than many proof-of-concept contact lens studies. However, the device still requires further validation over longer wearing periods and wider glucose ranges, especially during hypoglycemia and rapid glucose changes.

In addition to contact lens and ocular insert systems, microfluidic and eye-adjacent platforms have been developed for multiplexed tear analysis. Wang et al. reported an artificial intelligence-assisted wearable microfluidic colorimetric sensor system for the simultaneous monitoring of vitamin C, H^+^, Ca^2+^, and proteins in tears [105]. The system used a flexible PDMS microfluidic patch to collect approximately 20 μL of tears and a smartphone-based cloud server to process colorimetric data. The deep-learning CNN–GRU model corrected errors caused by pH variation and environmental color temperature, achieving $R^{2}$ values of 0.998 for pH prediction and 0.994 for the other biomarkers. The color reaction stabilized after approximately 1 min, and real tear testing was demonstrated using samples from five healthy volunteers. This platform shows how artificial intelligence can improve colorimetric tear sensing, but the study still needs larger clinical validation and disease-specific testing before diagnostic use. Overall, tear-based biosensors show strong promise for noninvasive monitoring of diabetes, glaucoma, dry-eye disease, renal dysfunction, and other ocular or systemic conditions. However, the field still faces several translational barriers. First, tear biomarker concentrations are generally low and can fluctuate due to tear dynamics, blinking, and reflex tearing. Second, contact lens sensors must balance sensing performance with oxygen permeability, optical transparency, softness, and long-term comfort. Third, enzymatic glucose sensors may suffer from enzyme degradation, drift, and biofouling from proteins and lipids in tears. Fourth, many devices are validated only in artificial tears, animal models, or small pilot studies. Future tear-based biosensors should therefore report standardized performance metrics, including LOD, sensing range, sensitivity, response time, selectivity, stability, wireless operating distance, biocompatibility, sample size, and agreement with blood or clinical reference methods. This type of performance-based reporting will make tear biosensors easier to compare and will support their transition from laboratory prototypes to clinically reliable wearable diagnostic systems.

### 4.3. Saliva-Based Biosensors

Recent developments in oral cavity biosensing have shown the feasibility of noninvasive and continuous chemical monitoring using saliva as a diagnostic medium. Saliva offers a painless and easily accessible route for tracking biomarkers such as lactate, glucose, uric acid, cortisol, and electrolytes; however, its diagnostic interpretation requires careful validation because salivary biomarker levels can be affected by salivary flow rate, food intake, oral hygiene, pH variation, enzyme activity, and local contamination. Therefore, saliva-based biosensors should be evaluated not only by their sensing mechanism, but also by key performance metrics such as detection range, sensitivity, response stability, selectivity, calibration reliability, and real-sample validation. The integration of biosensors into mouthguard and tooth-mounted platforms has transformed conventional saliva testing into real-time wearable bioanalytics with wireless communication, but long-term in-mouth stability and clinical correlation remain important challenges [106]. Saliva-based biosensors are also strongly affected by diet and oral environmental conditions. Food and drink intake can change salivary pH, glucose, lactate, electrolytes, proteins, enzymes, and oral microbial activity, which may directly alter biosensor readings. Carbohydrate- and sugar-rich foods can promote acid production by oral bacteria, lowering local pH and changing the electrochemical or optical response of pH-sensitive and enzyme-based sensors. In addition, salivary flow rate changes during eating, drinking, chewing, and stimulation, which can dilute or concentrate target biomarkers. Oral hygiene practices, including toothbrushing and mouth rinsing, may also affect salivary protein levels, microbial load, and surface contamination near intraoral sensors. Therefore, saliva-based wearable studies should clearly report whether measurements were performed under fasting, post-meal, resting, or stimulated conditions. For clinically meaningful continuous monitoring, future oral biosensors should include diet-aware calibration, pH correction, oral-hygiene control, and validation against standard saliva or blood-based reference assays [94,107,108].

A mouthguard-based uric acid biosensor was among the first fully integrated oral devices for dynamic in-mouth monitoring [23]. The system incorporated miniaturized electronics, including an amperometric sensing interface, potentiostat, microcontroller, and Bluetooth Low Energy transceiver, enabling real-time salivary uric acid detection as a possible proxy for blood uric acid. From a performance perspective, the uricase-modified mouthguard sensor showed a linear response to uric acid with a sensitivity of 2.32 μA/mM and $R^{2}=0.998$ in artificial saliva. The device also showed stable operation for about 4 h, with only small variation in the relative current response, suggesting acceptable short-term resistance to biofouling in saliva. These values support its potential for monitoring hyperuricemia, gout, and renal dysfunction. However, the system still requires stronger validation in larger human cohorts because salivary uric acid may be influenced by hydration, oral conditions, diet, and individual metabolic variability. Similar mouthguard-based platforms were later developed for salivary glucose sensing using glucose oxidase-modified electrodes and wireless telemetry [109]. Arakawa et al. developed a detachable mouthguard glucose biosensor on a dental PETG support using platinum and Ag/AgCl electrodes with a glucose oxidase membrane. The sensor detected glucose in artificial saliva over a range of 5 to 1000 μM/L, which covers the typical salivary glucose range reported for healthy and diabetic individuals. In phosphate-buffered solution, the sensor also showed a dynamic response over 1 to 1000 μM/L. The integrated wireless system achieved stable real-time monitoring for more than 5 h using a phantom jaw model, demonstrating the feasibility of continuous in-mouth glucose monitoring. Despite this progress, salivary glucose monitoring remains challenging because saliva glucose does not always directly reflect blood glucose, and sensor performance may be affected by enzyme degradation, pH changes, oral temperature, food residues, and biofouling during long-term use.

Beyond metabolic biomarkers, tooth-mounted and intraoral sensors have been explored for multiparametric biochemical and dietary monitoring. Tseng et al. introduced a passive radiofrequency trilayer tooth-mounted sensor composed of an active interlayer placed between two reverse-facing split-ring resonators [110]. The sensor was reported to be very small, down to approximately 2 mm × 2 mm, and could be attached to tooth enamel for in vivo monitoring during food and fluid intake. By using porous silk films and responsive PNIPAM hydrogel interlayers, the sensor could respond to changes in the dielectric properties of oral fluids associated with alcohol, salinity, sugar content, pH, and temperature. This design is attractive because it does not require a battery and can provide wireless readout. However, its output is based on RF resonance shifts rather than highly selective molecular recognition. Therefore, further improvement is needed to enhance analyte specificity, reduce cross-interference among food components, and establish quantitative calibration under complex oral conditions. For electrolyte monitoring, Lee et al. developed a stretchable, long-range wireless intraoral telemetry system for real-time sodium-intake tracking [111]. The platform used ultrathin, soft, and biocompatible hybrid electronics integrated with a sodium-selective sensing element and wireless readout. The device was tested in human subjects and demonstrated wireless sodium monitoring during food consumption, with communication to a smartphone or external device over a distance of up to about 10 m. This platform is relevant for dietary assessment and hypertension management because excessive sodium intake is directly associated with cardiovascular risk. However, in-mouth sodium measurement can be affected by saliva dilution, chewing behavior, food texture, incomplete extraction of sodium from solid foods, and sensor placement. Therefore, sodium-intake monitoring requires subject-specific calibration and stronger validation against standardized dietary sodium measurements.

Overall, the progress from uric acid and glucose mouthguard biosensors to multifunctional tooth-mounted and sodium-selective oral platforms highlights the growing role of saliva as a noninvasive diagnostic medium for personalized healthcare. Nevertheless, the current literature shows that most saliva-based wearable biosensors are still at the proof-of-concept or pilot-validation stage. Future studies should report standardized performance metrics, including limit of detection, sensitivity, response time, selectivity, operating range, short-term and long-term stability, resistance to biofouling, wireless operating distance, sample size, and comparison with blood or laboratory-standard assays. Such performance-based reporting will strengthen the clinical value of saliva-based biosensors and help move the field from wearable prototypes toward reliable diagnostic systems.

## 5. Self-Powered Biosensors

Self-powered wearable biosensors have emerged as a promising solution to the energy limitations of continuous and long-term health monitoring systems. These devices harvest energy from body motion, biochemical fuels in biofluids, or ambient sources such as light. As a result, they can reduce dependence on rigid batteries and support more autonomous, skin-conformal, and unobtrusive monitoring platforms [112,113]. However, the value of these systems should not be judged only by whether they eliminate batteries. Their practical performance should also be evaluated using power density, sensor sensitivity, analytical range, response time, stability under mechanical deformation, calibration reliability, and validation in real human samples.

**Figure 7:**
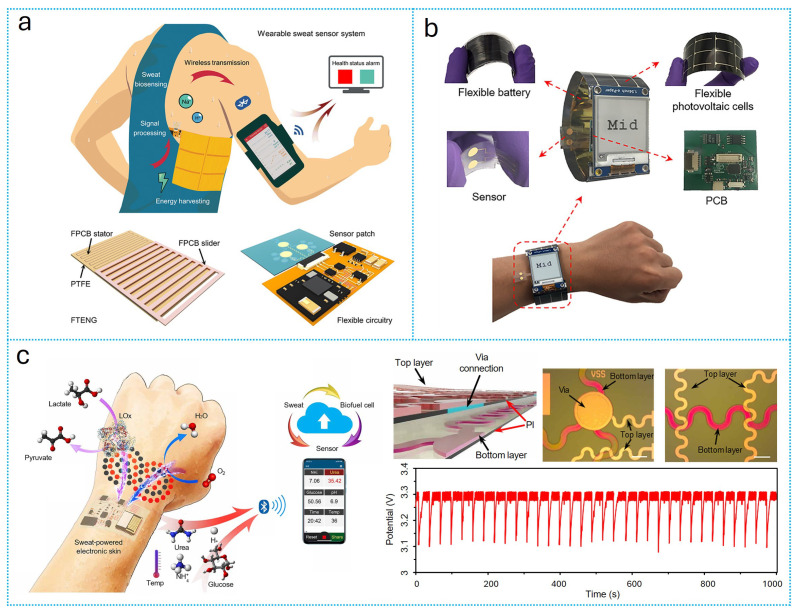
Fully integrated self-powered, battery-free wireless wearable biosensing systems: (**a**) A wireless wearable sweat biosensor powered by human motion using a flexible triboelectric nanogenerator (FTENG), enabling real-time, on-body analysis of sweat biomarkers for personalized healthcare. Reproduced with permission [114]. (**b**) A solar-cell-powered sweat glucose sensor integrated with a low-power electronic ink (e-ink) display. Reproduced with permission [115]. (**c**) A biofuel-powered soft electronic skin capable of multiplexed wireless biochemical sensing and human–machine interfacing. Reproduced with permission [116].

One prominent self-powered approach is based on triboelectric nanogenerators (TENGs), which convert mechanical energy from body motion into electrical energy through contact electrification and electrostatic induction. Song et al. demonstrated a fully integrated, battery-free wearable platform consisting of a flexible TENG (FTENG) array, a microfluidic sweat-sensing patch, and a power management circuit, as illustrated in Figure 7a [114]. The FTENG produced a maximum power output of 0.94 mW, corresponding to a power density of approximately 416 mW m^−2^, at a load resistance of 4.7 MOhm. The device also showed frequency-dependent short-circuit currents of 8.39, 19.11, and 42.25 microA at 0.5, 1.25, and 3.3 Hz, respectively, indicating that motion intensity directly affects the available power. From the sensing perspective, the integrated pH and Na^+^ sensors showed near-Nernstian sensitivities of 56.28 mV/pH and 58.63 mV per decade, respectively, over physiologically relevant ranges of pH 4–8 and Na^+^ concentrations of 12.5–200 mM. The Na^+^ sensor reached a new stable reading in about 2 min when the concentration changed from 50 to 200 mM at a flow rate of 2 μL/min. The system-level power consumption was also carefully matched to the harvester output; each wireless measurement cycle consumed an average current of about 330 microA for approximately 510 ms. During on-body treadmill running, the system achieved up to 18 operation cycles within 60 min, with charging/discharging intervals of 2.1–3.7 min. These results demonstrate real-world feasibility, but they also reveal an important limitation: the measurement frequency depends on the amount and regularity of body motion.

Photovoltaic energy harvesting represents another strategy for supporting wearable sensing under ambient or solar illumination, as shown in Figure 7b. Zhao et al. reported a fully integrated self-powered smartwatch for continuous sweat glucose monitoring, where flexible photovoltaic cells charged Zn–MnO_2_ batteries that powered an enzymatic glucose sensor and an electronic ink display [115]. The glucose sensor was calibrated over a sweat-relevant range of 50–200 microM and showed an average sensitivity of 3.29 nA microM^−1^, with a relative standard deviation of 8.8%. After 2 h of continuous measurement, the sensitivity remained 3.18 nA microM^−1^, corresponding to only 3.3% variation. The sensor also retained average sensitivities of 3.08 and 3.10 nA microM^−1^ after 30 bending cycles and 30 skin-contact/friction events, respectively. At the system level, the current detection resolution was 6 nA, corresponding to approximately 2 microM glucose. The energy module could be charged to 6.0 V within 1 h under outdoor sunlight and supported operation for up to 8 h; under room light, it required about 2 h to charge to 4.2 V and supported approximately 1 h of operation. These values demonstrate good integration between energy harvesting, storage, sensing, and display. Nevertheless, this approach remains dependent on light availability, and the displayed output was mainly qualitative, using low, medium, and high glucose-level indicators rather than direct concentration values. Therefore, future solar-powered glucose systems should include stronger quantitative calibration and clearer clinical correlation between sweat and blood glucose levels.

Biofuel cells (BFCs) provide another self-powered route by converting biochemical fuels in sweat, such as glucose and lactate, into electrical energy. A representative example is the perspiration-powered electronic skin (PPES) developed by Yu et al., which integrated lactate BFCs with multiplexed biochemical sensors for glucose, pH, urea, ${\mathrm{NH}}_{4}^{+}$, and skin temperature, as illustrated in Figure 7c [116]. The PPES achieved a power density of 3.5 mW cm^−2^ in untreated human sweat and maintained stable performance during 60 h of continuous operation. It also enabled Bluetooth-based wireless transmission during prolonged physical activity. From a performance-evaluation perspective, this system is important because it reports not only power generation but also real-sample operation, multi-analyte sensing, and long-duration stability. However, BFC-based platforms remain sensitive to enzyme stability, oxygen availability, sweat-fuel concentration, and biofouling at the electrode surface. These factors can gradually reduce output current and introduce drift in both energy harvesting and sensing accuracy.

The dual role of BFCs as both power sources and sensing elements has also been demonstrated using textile-based platforms. Jeerapan et al. developed stretchable textile BFCs using screen-printed, stress-enduring inks and serpentine electrode patterns [117]. The glucose and lactate BFCs generated maximum power densities of approximately 160 and 250 microW cm^−2^, respectively, with open-circuit voltages of 0.44 and 0.46 V. Importantly, the devices maintained stable power output after repeated mechanical deformation, including 100 cycles of 100% stretching, and were demonstrated on human subjects. These results show that mechanical durability is as important as analytical sensitivity for wearable self-powered biosensors. Still, the long-term use of enzyme-based BFCs is limited by enzyme degradation, changing sweat composition, and the need for repeated calibration under different physiological and environmental conditions.

Hybrid energy harvesting strategies can improve power reliability by combining complementary energy sources, such as BFCs, TENGs, piezoelectric nanogenerators, photovoltaic cells, and supercapacitors [118]. For example, systems that combine sweat-based BFCs with supercapacitors can store biochemical energy and provide a more stable output than BFCs alone. Similarly, TENG–BFC hybrid systems can use motion-derived power during activity and sweat-metabolite-derived power during perspiration. These designs are promising because they reduce the weakness of single-source harvesters. However, they also introduce additional design complexity, including charge management, impedance matching, flexible interconnection, and long-term encapsulation. Therefore, hybrid systems should be evaluated not only by peak output power but also by continuous operating time, data transmission frequency, calibration stability, and performance after repeated bending, washing, sweating, and skin contact.

Despite these advances, several barriers remain before self-powered wearable biosensors can be translated into clinically reliable tools. First, sweat composition varies with sweat rate, hydration status, skin location, temperature, and individual physiology, which can affect both energy harvesting and analyte measurement. Second, calibration drift remains a major concern, especially for ion-selective electrodes, enzymatic electrodes, and BFC-based sensors. Third, biofouling from proteins, salts, skin debris, and oxidation products can block active electrode sites and degrade sensitivity over time. Fourth, many studies report excellent laboratory performance but still include limited human-subject testing and short monitoring periods. To improve translational value, future studies should report a common performance set that includes limit of detection or analytical range, sensitivity, response time, selectivity, mechanical durability, storage stability, on-body operating time, sample size, real-sample validation, and calibration strategy. Such reporting would make self-powered biosensors easier to compare and would directly support their movement from proof-of-concept demonstrations toward clinically useful wearable diagnostic systems.

### 5.1. Evaluation of Current Wearable Biofluid Biosensors

Although wearable biofluid biosensors have shown strong potential for continuous and non-invasive health monitoring, their practical translation remains limited by gaps between laboratory performance and real-world reliability. As summarized in Table 2, current devices are commonly evaluated using performance metrics such as limit of detection, sensing range, sensitivity, response stability, selectivity, calibration reliability, and validation in real biological samples. However, high sensitivity or low detection limit alone is not sufficient for clinical use. Sweat rate, sampling location, skin contamination, evaporation, motion artifacts, temperature fluctuation, and inter-individual physiological variation can significantly alter biomarker concentration and sensor response [17,61,78]. Therefore, practical evaluation must combine analytical performance with long-term stability, reproducibility, calibration robustness, and paired biofluid–blood validation.

The performance matrix shows that different biomarkers face different translational limitations. Glucose sensors can detect sweat glucose within low micromolar ranges, but their clinical value remains limited by weak or variable blood–sweat correlation, making continuous diabetes monitoring difficult without strong calibration. Lactate sensors can achieve very low detection limits and high sensitivity, yet saturation at higher concentrations and slower response times may limit their use during intense exercise or rapid metabolic changes. Uric acid, tyrosine, and cortisol sensors show promising analytical sensitivity, but their broader clinical adoption is still restricted by limited sample size, antibody dependence, incubation needs, and insufficient validation under continuous on-body conditions [13,15,119,120].

For electrolyte and multiplexed sensing, the main challenge is not only detection, but stable interpretation during daily wearable use. Sodium and potassium sensors generally provide near-Nernstian potentiometric responses over sweat-relevant ranges, but calibration drift, sweat-rate dependence, and cross-subject variability remain major barriers [57]. Similarly, advanced multiplexed platforms using molecularly imprinted polymers, nanomaterials, or affinity-based interfaces can monitor multiple amino acids, vitamins, metabolites, and lipids, but device complexity, matrix interference, reproducibility, and large-scale clinical validation remain unresolved [41]. These findings indicate that recognition chemistry and material design should be selected not only for sensitivity, but also for selectivity, durability, manufacturability, and compatibility with continuous wearable operation [33,121].

Overall, Table 2 indicates that the major barrier in wearable biofluid biosensors is no longer simple biomarker detection, but the development of clinically reliable systems that can operate accurately across users, environments, and time. Future studies should therefore prioritize standardized benchmarking, paired biofluid–blood studies, long-term on-body testing, anti-biofouling strategies, scalable fabrication, and large-scale clinical validation. Reporting common performance metrics, including LOD, sensing range, sensitivity, response time, selectivity, stability, wireless operating performance, sample size, and calibration strategy, will make wearable biosensors easier to compare and support their transition from promising laboratory prototypes to dependable healthcare technologies.

### 5.2. Recent Developments in AI Integration, Advanced Nanomaterials, and Clinically Validated Wearable Biosensors

Recent progress in wearable biosensing has moved beyond simple biomarker detection toward integrated systems that combine advanced materials, wireless communication, artificial intelligence, and clinical validation. This shift is important because wearable biosensors generate continuous and complex data streams that cannot always be interpreted through single calibration curves. Therefore, current research increasingly emphasizes complete system performance, including sensing accuracy, user-specific calibration, data interpretation, long-term stability, and clinical reliability.

Artificial intelligence has become an important tool for improving wearable biosensor interpretation by supporting signal correction, anomaly detection, personalized calibration, and prediction of physiological or disease states from noisy wearable data [122]. However, AI-assisted biosensors require high-quality datasets, diverse user populations, explainable models, privacy protection, and clinical validation to ensure reliable real-world performance. For example, an AI-assisted wearable microfluidic colorimetric sensor system combined a flexible tear-collection patch, smartphone imaging, cloud-based analysis, and CNN–GRU models to monitor vitamin C, H^+^, Ca^2+^, and proteins in tears [105]. The system achieved test-set $R^{2}$ values of 0.998 for pH prediction and 0.994 for the other biomarkers using approximately 20 microL of tears, showing that AI can correct colorimetric errors caused by pH variation and environmental color temperature. However, larger clinical studies are still required to confirm diagnostic reliability.

Advanced nanomaterials have also improved wearable biosensor performance by increasing active surface area, enhancing electron transfer, improving flexibility, and lowering detection limits. Materials such as laser-engraved graphene, laser-induced graphene, graphene–silver nanowire hybrids, metal–organic frameworks, carbon nanofibers, noble metal nanoparticles, hydrogels, and molecularly imprinted polymers have been widely used for this purpose. For example, laser-engraved graphene-based sweat sensors have enabled sensitive detection of uric acid and tyrosine, while graphene–silver nanowire hybrid structures have supported transparent and stretchable contact lens sensors for tear glucose and intraocular pressure monitoring [15,101]. Similarly, MOF-derived carbon nanofiber composites and MIP-based regenerable electrodes are promising for multiplexed sweat analysis because they provide high surface area and improved recognition capability [41,100]. However, nanomaterial-based platforms still face challenges related to reproducibility, large-scale fabrication, biocompatibility, long-term stability, and sensor-to-sensor variability. Clinically validated systems remain less common than laboratory prototypes, but the NovioSense conjunctival fornix sensor showed progress by achieving a mean absolute relative difference of 16.7, median absolute relative difference of 13.3, and 99.7% of points in Clarke error grid A+B regions in patients with type 1 diabetes [14]. Overall, future studies should report both analytical and translational metrics, including detection limit, sensitivity, response time, stability, sample size, real-sample testing, data-processing method, and comparison with clinical reference standards.

## 6. Conclusions

This review presents an overview of recent advances in non-invasive wearable biosensing technologies, with a focus on biofluid-based systems for chronic disease monitoring. By examining biofluids such as sweat, tears, and saliva, this work highlights their diagnostic value, easy accessibility, and ability to support continuous, real-time health monitoring outside traditional clinical settings. The roles of biorecognition elements, signal transduction mechanisms, and data acquisition strategies are discussed to explain how biochemical signals are converted into useful physiological information. Recent progress in materials science, microfluidics, and system integration has enabled flexible, skin-conformal, and portable wearable biosensors. These devices can monitor a wide range of biomarkers related to metabolism, hydration, stress, and immune function. Improvements in sweat collection methods, including passive, active, and microfluidic-assisted approaches, have enhanced sampling reliability and measurement accuracy under different physiological conditions. In addition, the integration of wireless communication and artificial intelligence-based data analysis has turned wearable biosensors into intelligent monitoring systems that provide personalized feedback and support predictive healthcare. Despite these advances, several challenges remain that limit large-scale adoption and clinical use. These challenges include variability in biofluid composition, limited sensor sensitivity and reproducibility, long-term stability issues, biofouling, and the lack of standardized calibration and validation methods. Overcoming these limitations will require improvements in device design, materials, data analysis methods, and large-scale clinical validation to ensure reliable and clinically approved wearable biosensing systems.

## 7. Future Directions

The future of wearable biosensors lies in addressing key challenges related to sensor accuracy, multi-analyte integration, and real-time data analysis. Key areas of development include:

1. Improving Accuracy and Sensitivity: To enhance sensor reliability, future research must focus on minimizing calibration drift and signal degradation. Advances in flexible substrates and nanomaterials will be crucial for maintaining accuracy during prolonged use.
2. Multi-Analyte Platforms: Developing biosensors capable of simultaneously detecting multiple biomarkers (e.g., glucose, lactate, electrolytes) in biofluids such as sweat and saliva will enable more comprehensive health monitoring. The integration of microfluidics and multiplexed sensing techniques will be central to this progress.
3. AI Integration for Personalized Monitoring: Machine learning algorithms can be leveraged to analyze complex datasets generated by wearable biosensors. Artificial intelligence (AI) can enable real-time health monitoring, predictive analytics, and personalized interventions, particularly in chronic disease management. AI-driven sensor calibration may further improve data accuracy without manual intervention.
4. Clinical Validation and Data Standardization: Clinical trials are essential for validating the performance of wearable biosensors in real-world settings. Standardized testing protocols and seamless data integration with electronic health records (EHRs) will be necessary for widespread clinical adoption.
5. Wearability and Comfort: Long-term wearability remains a challenge, particularly with respect to skin comfort and sensor durability. Advances in materials science, including soft, stretchable electronics and biocompatible hydrogels, will enhance user comfort during extended wear.
6. Affordability and Accessibility: To maximize global impact, future wearable biosensors must be cost-effective and accessible to diverse populations, particularly in low-resource settings. Collaboration with global health initiatives could facilitate widespread adoption of these technologies.

## Figures and Tables

**Table 2:** Comparative performance of representative wearable biofluid biosensors for non-invasive monitoring.

Biofluid	Biomarker	Detection Method/Platform	Key Numerical Performance	Main Limitation	Ref.
Sweat	Glucose	Cotton/polypyrrole/Cu/ Cu–Mn fabric sensor; DPV electrochemical sensing	LOD: 125 μM; LOQ: 378 μM; range: 50–400 μM; $R^{2}$ = 0.983; validated in human sweat	Needs stronger blood–sweat correlation and continuous diabetes-monitoring validation	[119]
Sweat	Lactate	Lactate oxidase-functionalized organic electrochemical transistor	Detectable response down to 11 nM; tested from 0.01 to 9.8 mM; sensitivity up to 1.9 mA/mM; response time about 5–10 min; device lifetime about 40 min	Saturates above about 1 mM; limited lifetime restricts long-term exercise monitoring	[120]
Sweat	Glucose, lactate, Na^+^, K^+^	Fully integrated multiplexed electrochemical patch	Glucose sensitivity: 2.35 nA/μM; lactate sensitivity: 220 nA/mM; Na^+^ sensitivity: 64.2 mV/decade; K^+^ sensitivity: 61.3 mV/decade; Na^+^ range: 10–160 mM; K^+^ range: 1–32 mM	Requires careful calibration because sweat rate, temperature, and exercise intensity affect signal output	[82]
Sweat	Na^+^, K^+^	Solid-contact ion-selective electrodes using PEDOT or POT	Na^+^ sensitivity: 52.4–56.4 mV/decade; K^+^ sensitivity: 45.7–54.3 mV/decade; on-body exercise trials up to 90 min	Calibration drift, sweat-rate dependence, and cross-subject variability remain important challenges	[57]
Sweat	Uric acid/ tyrosine	Laser-engraved graphene electrochemical sensor with microfluidic sweat sampling	UA LOD: 0.74 μM; Tyr LOD: 3.6 μM; UA sensitivity: 3.50 mA per μM per cm^2^; Tyr sensitivity: 0.61 mA per μM per cm^2^	Clinical sample size remains limited; diagnostic thresholds require larger cohort validation	[15]
Sweat	Cortisol	Graphene-based wireless immunosensor/aptamer-type sweat cortisol platform	Reported low-level cortisol detection with wireless sweat monitoring; some platforms report pg/mL-level detection and validation against reference assays	Hormone sensing is affected by low analyte abundance, nonspecific adsorption, sweat-rate variation, and calibration against blood or saliva cortisol	[13,99]
Sweat	pH and Na^+^	Battery-free TENG-powered sweat sensor system	Maximum TENG power: 0.94 mW; power density: 416 mW per m^2^; pH sensitivity: 56.28 mV/decade; Na^+^ sensitivity: 58.63 mV/decade; Na^+^ response time about 2 min	Measurement frequency depends on body-motion intensity and regularity	[114]
Sweat	Na^+^, Cl^−^, glucose	Autonomous iontophoretic sweat extraction wristband	Na^+^ sensitivity: 63.2 mV/decade; Cl^−^ sensitivity: 55.1 mV/decade; NaCl range: 10–160 mM; glucose sensitivity: 2.1 nA/μM; glucose range: 0–100 μM	Repeated stimulation may cause discomfort; pilocarpine response and local sweat composition vary between users	[83]
Sweat	Glucose	Self-powered smartwatch sweat glucose sensor	Circuit current resolution: 6 nA; approximately equivalent to 2 μM glucose; charged to 6.0 V within 1 h under outdoor sunlight; operating duration up to 8 h	Sweat glucose still requires stronger correlation with blood glucose before clinical use	[115]
Sweat	Amino acids/ vitamins	Regenerable graphene electrodes with molecularly imprinted polymers	Multiplexed monitoring of amino acids, vitamins, metabolites, and lipids; repeated in situ regeneration demonstrated during rest and exercise testing	Complex device design; analyte-specific calibration and large-scale clinical validation are still needed	[41]
Saliva	Uric acid	Wearable mouthguard biosensor with integrated wireless electronics	Designed for real-time salivary uric acid monitoring; covers healthy and hyperuricemia-relevant salivary uric acid ranges	Diet, oral contamination, salivary flow rate, and oral hygiene can affect readings	[23]
Saliva	Glucose	Mouthguard glucose biosensor with telemetry system	Artificial saliva detection range: 5–1000 μM/L; stable wireless monitoring for more than 5 h in a phantom jaw model	Human validation and reliable saliva–blood glucose calibration remain necessary	[109]
Saliva	Na^+^	Stretchable intraoral wireless sodium sensor	Continuous sodium-intake tracking demonstrated in human subjects with wireless oral telemetry	Diet, saliva dilution, oral movement, and comfort can affect long-term monitoring	[111]
Tear	Glucose and intraocular pressure	Transparent graphene–AgNW smart contact lens	Optical transparency more than 91 percent; stretchability about 25 percent; demonstrated rabbit tear glucose monitoring and bovine-eye pressure testing	Human tear glucose correlation and long-term comfort require further validation	[101]
Tear	Glucose	Battery-free soft smart contact lens with wireless power and LED display	Wireless real-time visualization of tear glucose response demonstrated in rabbit model	Needs human validation and improved calibration for daily glucose variability	[103]
Tear	Glucose	Photonic hydrogel sensor attached to commercial contact lens	Sensitivity: 12 nm/mM; saturation time less than 30 min; smartphone-based optical readout	Optical response can be affected by lighting, alignment, tear volume, and evaporation	[104]
Tear	Glucose	NovioSense conjunctival fornix sensor	Clinical diabetic trial reported MARD about 16.7 percent and MedARD about 13.3 percent; high Clarke error-grid agreement reported	Accuracy still needs improvement before replacing conventional glucose monitoring	[14]

## Data Availability

Data sharing is not applicable to this article as no new data were created or analyzed in this study.
